# Integrative Multi‐Omics and Routine Blood Analysis Using Deep Learning: Cost‐Effective Early Prediction of Chronic Disease Risks

**DOI:** 10.1002/advs.202412775

**Published:** 2025-04-02

**Authors:** Zhibin Dong, Pei Li, Yi Jiang, Zhihan Wang, Shihui Fu, Hebin Che, Meng Liu, Xiaojing Zhao, Chunlei Liu, Chenghui Zhao, Qin Zhong, Chongyou Rao, Siwei Wang, Suyuan Liu, Dayu Hu, Dongjin Wang, Juntao Gao, Kai Guo, Xinwang Liu, En Zhu, Kunlun He

**Affiliations:** ^1^ Medical Innovation Research Division Chinese PLA General Hospital Beijing Key Laboratory of Chronic Heart Failure Precision Medicine Chinese PLA General Hospital Medical Engineering Laboratory of Chinese PLA General Hospital Beijing 100038 China; ^2^ School of Computer science National University of Defense Technology Trinity Avenue, Kaifu District Changsha Hunan 410005 China; ^3^ Department of Cardiovascular Surgery Nanjing Drum Tower Hospital Chinese Academy of Medical Science & Peking Union Medical College Nanjing 210000 China; ^4^ West China School of Basic Medical Sciences & Forensic Medicine Sichuan University Chengdu 610000 China; ^5^ Department of Cardiology Hainan Hospital of Chinese People's Liberation Army General Hospital Sanya 572000 China; ^6^ Center for Synthetic & Systems Biology Tsinghua University Beijing 100084 China; ^7^ Department of Psychiatry The First Affiliated Hospital of Chongqing Medical University Chongqing 400010 China; ^8^ Key Laboratory of Major Brain Disease and Aging Research (Ministry of Education) Chongqing Medical University Chongqing 400010 China

**Keywords:** multi‐omics, pathway discovery, potential risk classification

## Abstract

Chronic noncommunicable diseases (NCDS) are often characterized by gradual onset and slow progression, but the difficulty in early prediction remains a substantial health challenge worldwide. This study aims to explore the interconnectedness of disease occurrence through multi‐omics studies and validate it in large‐scale electronic health records. In response, the research examined multi‐omics data from 160 sub‐healthy individuals at high altitude and then a deep learning model called Omicsformer is developed for detailed analysis and classification of routine blood samples. Omicsformer adeptly identified potential risks for nine diseases including cancer, cardiovascular conditions, and psychiatric conditions. Analysis of risk trajectories from 20 years of large clinical patients confirmed the validity of the group in preclinical risk assessment, revealing trends in increased disease risk at the time of onset. Additionally, a straightforward NCDs risk prediction system is developed, utilizing basic blood test results. This work highlights the role of multiomics analysis in the prediction of chronic disease risk, and the development and validation of predictive models based on blood routine results can help advance personalized medicine and reduce the cost of disease screening in the community.

## Graphical abstract

Omicsformer, a deep learning model, integrates multi‐omics and routine blood data to accurately predict risks for nine chronic diseases, including cancer and cardiovascular conditions. Validated using large scale clinical data, it reveals early risk trajectories, advancing personalized medicine and offering a cost‐effective, community‐based solution for chronic disease prediction and prevention.

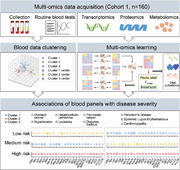

## 1 Introduction

Chronic noncommunicable diseases (NCDs) pose a significant challenge to global health. The prevalence of NCDs is primarily influenced by genetic, environmental, and medical factors, among others. According to the World Health Statistics 2023, major NCDs–such as cardiovascular disease, cancer, chronic respiratory disease, and diabetes–accounted for 74% of global deaths and 63% of global disability‐adjusted life‐years (DALYs).^[^ ^1^, ^2^ ^]^ Early detection and screening of these diseases are crucial for reducing societal costs and alleviating the burden on patients.^[^ ^3^ ^]^ Primary healthcare institutions, particularly community hospitals, play a vital role in facilitating essential screening.^[^ ^4^ ^]^ In the context of large‐scale community screenings and health assessments at medical centers, identifying an effective disease warning system suitable for large populations is critical.^[^ ^5^ ^]^ Compared to imaging techniques, hematological screening offers a more convenient, efficient, and cost‐effective method of examination, providing insights into an individual's health status during a given period. However, specific biomarkers developed for tumors and immune diseases often cannot be widely used due to their high specificity and associated costs.^[^ ^6^ ^]^ Thus, it is of considerable importance to further investigate the potential of routine hematological examinations in predicting the onset of NCDs.

Existing studies often struggle to effectively characterize the pre‐onset state of NCDs.^[^ ^7^ ^]^ Sub‐health is defined as a condition that exists between optimal health and NCDs, characterized by diminished vitality, functionality, and adaptability over time. This sub‐health status is influenced by various factors, including environment, rest, diet, and mood.^[^ ^8^ ^]^ Current research indicates that in plateau environments, low atmospheric pressure and reduced oxygen levels can adversely affect metabolic processes and organ function during the body's adaptation.^[^ ^9^ ^]^ The inclusion of high‐altitude populations is particularly valuable because adaptation to high‐altitude conditions activates metabolic pathways and the immune system, which can help uncover early pathological processes that are difficult to detect under normoxic conditions. Metabolic diseases, tumors, cardiovascular and cerebrovascular disorders, as well as degenerative conditions, are chronic diseases closely associated with hypoxic signaling pathways, posing significant threats to health.^[^ ^10^, ^11^ ^]^ Hypoxia may activate immune and metabolic pathways, with the upregulation of hypoxia‐inducible factors and reactive oxygen species (ROS) potentially increasing the risk of cancer and other diseases.^[^ ^12^ ^]^ Hypoxia is a common feature of most solid tumors, and when tumors exceed a certain size, their internal environment becomes hypoxic. This low oxygen state significantly influences tumors through the hypoxia‐inducible factor (HIF) signaling pathway.^[^ ^13^ ^]^ Under hypoxic conditions, lung cancer cells exhibit altered biological characteristics, including reduced sensitivity to radiotherapy and chemotherapy, inhibition of apoptosis, changes in the cell cycle, enhanced angiogenic potential, and increased invasiveness and metastatic capacity.^[^ ^14^ ^]^ Additionally, hypoxia promotes stemness in liver cancer cells.^[^ ^15^ ^]^ Furthermore, beyond oncology, hypoxia holds substantial significance in various other medical fields. The elevated release of norepinephrine and excitatory amino acids may contribute to an increased risk of cardiovascular and neurological disorders.^[^ ^16^, ^17^ ^]^ In cardiovascular diseases, hypoxia can lead to ischemic heart disease and heart failure,^[^ ^18^ ^]^ while in neurological conditions such as stroke and traumatic brain injury, hypoxia‐induced damage remains a leading cause of high morbidity and mortality.^[^ ^19^ ^]^ Thus, the extensive impact of hypoxia on human health underscores the necessity of studying hypoxia and its underlying molecular mechanisms. Hematological assessments of high‐altitude populations, who have adapted to hypoxic conditions, can reveal unique pathophysiological processes under these states, providing crucial insights into early, pre‐disease conditions that are otherwise elusive.^[^ ^20^ ^]^

The complexity intrinsic to biological systems often transcends the capabilities of single‐omics data, necessitating the synthesis and in‐depth analysis of multi‐omics information. Multi‐omics approach is instrumental in bridging knowledge gaps and unraveling the complex interactions within biological systems.^[^ ^21^, ^22^, ^23^, ^24^ ^]^ For instance, Kikuchi's application^[^ ^25^ ^]^ of quantitative proteomics, transcriptomics, and phosphorylation proteomics has shed light on the mechanisms of compensatory hypertrophy in renal proximal tubules. Similarly, a multi‐omics study involving gut microbiota, liver, and blood samples from hepatitis C patients has provided insights into metabolic perturbations along the gut‐liver axis.^[^ ^26^ ^]^ These comprehensive datasets are crucial for uncovering fundamental biological processes and identifying critical disease‐causing factors.^[^ ^27^ ^]^ Predominantly, omics research has been disease‐centric, with limited focus on early‐stage disease states. Addressing this gap is vital for reducing healthcare costs and enhancing overall health through early intervention.^[^ ^7^ ^]^

The emergence of Transformers^[^ ^28^ ^]^ and large‐scale model techniques has significantly advanced the field of multi‐omics integrative learning, characterized by sophisticated attention mechanisms and enhanced learning capabilities. For instance, Yang et al. developed a Transformer‐based framework, TransDriver,^[^ ^29^ ^]^ which integrates multi‐omics data to identify key genes that drive cancer. Additionally, Lin et al. employed multimodal deep learning algorithms for clustering single‐cell multi‐omics data, demonstrating the potential of leveraging deep learning in multi‐omics integration.^[^ ^30^ ^]^ Despite these advancements, significant challenges remain in thoroughly elucidating the etiology of diseases and the complex molecular networks that bridge different omics layers. As multi‐omics technologies continue to evolve, their application in large‐scale disease models is becoming increasingly prominent, particularly in the areas of disease prediction and classification.^[^ ^31^, ^32^, ^33^, ^34^ ^]^ For example, MOGONET^[^ ^35^ ^]^ effectively integrates multi‐omics data using graph convolutional networks (GCN),^[^ ^36^ ^]^ thereby improving disease classification accuracy. Furthermore, Cai et al. applied machine learning for cancer type classification,^[^ ^37^ ^]^ while Osseni et al. utilized multi‐omics data for precise cancer classification and prediction,^[^ ^38^ ^]^ highlighting the transformative potential of multi‐omics technologies in oncology. However, these approaches often underestimate the intricate interactions between different omics layers. Achieving a comprehensive understanding of disease processes and enhancing predictive model accuracy are crucial to the application of multi‐omics technologies in drug development, health assessment, and disease prediction. Notably, recent work by Gao et al. has integrated environmental, metabolomic, proteomic, and routine blood test data to construct a detailed environmental health network.^[^ ^39^ ^]^

In our study, we identified hypoxic adaptation as a representative pre‐disease state, and collected blood and urine samples from high‐altitude acclimated populations for multi‐omics analysis. Utilizing a multi‐omics aggregation approach based on Transformers, we detected changes in the omics profile associated with distinct patterns of routine blood tests and proposed a novel method for assessing individual health status based on these patterns. The validity and effectiveness of our approach were confirmed using a large clinical patient dataset, supplemented by prospective cohort studies. This pioneering research aims to leverage a simple test to predict high‐risk disease factors, enhance community screening capabilities for chronic diseases, facilitate early detection and treatment, and ultimately reduce average healthcare costs.

## 2 Results

### 2.1 Overall Framework of Article

**Figure 1:**
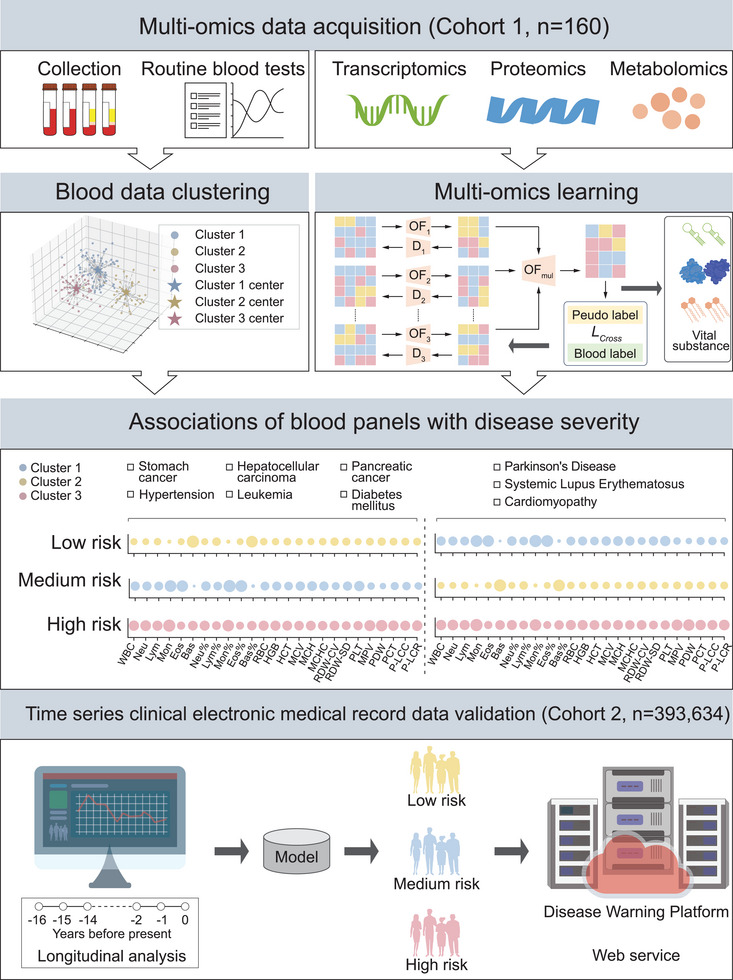
Flowchart of the overall framework of our article. Initially, we conducted sequencing and hematological sampling on a cohort comprising 160 healthy individuals. Subsequently, employing our clustering methodology, we categorized the hematological data into three distinct risk groups. Utilizing multi‐omics datasets, we classified these three risk groups and identified pertinent biomarkers. Subsequently, employing a battery of biological assays, we analyzed the identified biomarkers in conjunction with hematological parameters, resulting in the formulation of comprehensive hematological panels. These panels were subsequently validated using extensive time‐series clinical data and culminated in the development of a user‐friendly web server interface.

In our study, we developed a comprehensive framework, as illustrated in **Figure** **1**, compassing five essential stages: collection and sequencing of multi‐omics data, clustering and labeling of routine blood data, learning from multi‐omics data, discovering pathways across omics layers, determining the routine blood panel for different diseases, and validation with large‐scale clinical data. Initially, we collected and sequenced multi‐omics data by obtaining blood samples from 160 healthy individuals for comprehensive transcriptomics and metabolomics sequencing. Concurrently, we collected urine samples from the same cohort to acquire proteomic and metabolomic data. Routine blood tests were conducted on the blood samples at the same time. The unsupervised deep clustering algorithms were used to process phenotype data, extracting high‐level features and generating three distinct phenotype labels through an advanced clustering mechanism. Moreover, these phenotype labels were integrated with the amassed multi‐omics data, including transcriptomics, proteomics, and blood and urine metabolomics, to form training and testing sets. Here, transformer models were employed to learn the intricate relationships within and across different omics layers. Next, multi‐omics data were integrated to identify key substances and pathways within the omics layers. These analyses were combined to determine the corresponding routine blood panels for different diseases. The final stage focused on validating our blood panels using extensive clinical phenotype data. This involved applying the model to predict health statuses at specific time points and evaluating its precision against actual disease occurrences in the individuals at subsequent time points. This validation confirmed the accuracy of our blood panels in predicting health status, demonstrating their potential utility in early disease detection and monitoring. In addition, the data standards and definition of variables in the article are described in the Table S5 (Supporting Information).

### 2.2 Phenotypic Data and Multi‐Omics Presentation

**Figure 2:**
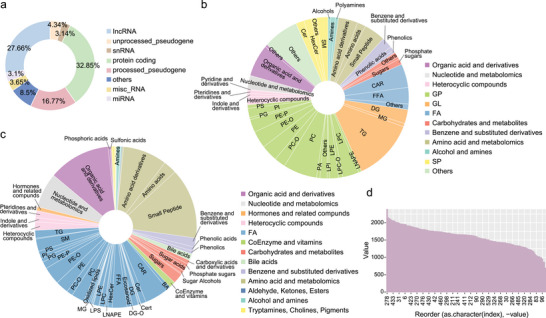
Detailed classification of the transcriptome, proteome, and metabolome sequencing data. a) Distribution of RNA sequence types in transcriptomic data; b) Comprehensive metabolomic profiling of blood samples; c) Comprehensive metabolomic profiling of urine samples; d) Protein abundance distribution in proteomic data.

High‐altitude populations exhibit significant phenotypic differences under hypoxic conditions, which are difficult to observe and distinguish in lowland populations. To further study the changes in the body under hypoxic conditions, In this study, we thoroughly collected blood and urine specimens from 160 high altitude acclimatized individuals, employing both routine blood testing and advanced multi‐omics sequencing techniques. The routine blood tests measured 25 distinct biochemical and hematological parameters, with the comprehensive results and multi‐omics sequencing data detailed in Data S1 (Supporting Information). A transcriptomic analysis of the blood samples revealed a diverse array of RNA species, as illustrated in **Figure** **2**a. We cataloged a total of 60 604 genes, with a predominance of protein‐coding genes constituting 32.85%, followed by long non‐coding RNAs (lncRNAs) at 27.66%, and processed pseudogenes at 16.77%. Additional categories included miscellaneous RNAs (misc‐RNAs) at 3.65%, small nuclear RNAs (snRNAs) at 3.14%, unprocessed pseudogenes at 4.34%, and microRNAs (miRNAs) at 3.1%, with other RNA types comprising 8.5% of the total. This chart not only underscores the diversity of RNA species within the blood samples but also emphasizes the significant representation of protein‐coding genes, which were subsequently selected for in‐depth analysis to unravel the intricacies of gene expression regulation in the cohort.

Metabolomic sequencing has yielded a detailed compositional analysis of the blood and urine samples from our study cohort, with the findings presented in Figure 2b,c. A total of 1826 metabolites were identified in blood, whereas urine analysis revealed 1380 distinct metabolites. The proportions of these metabolites are graphically represented in the charts, where the size of each segment correlates with the relative abundance of each metabolite category detected. Within these segments, specific metabolite types are delineated, encompassing various biochemical classes such as amino acids, organic acids, nucleotides, carbohydrates, hormones, coenzymes, and vitamins. This extensive metabolite profiling offers a panoramic view of the metabolic states of the participants, encapsulating the heterogeneity and complexity of their physiological processes. Then, the urine sample analysis identified 8143 proteins, with 3319 proteins consistently found in over 95% of the samples, indicating stable protein levels across the participants. Figure 2d shows that a few samples had very high protein levels, but most samples had moderate and consistent levels, demonstrating the reliability of our protein measurement methods. This consistent detection of proteins is essential for the accuracy of our multi‐omics analysis.

### 2.3 Blood Routine Clustering Results

**Figure 3:**
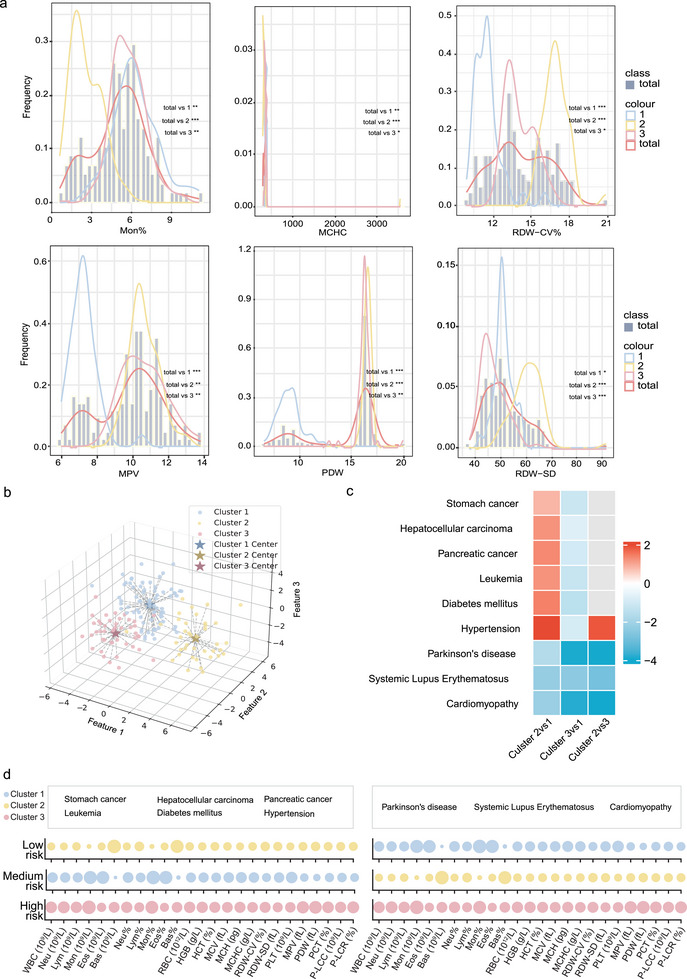
Use clinical blood routine and omics data to build a blood routine panel for healthy people. a) The probability density diagram of blood routine data of all populations and different cluster populations, total represents all populations, 1, 2, and 3 represents different cluster populations; b) Results of deep cluster analysis of clinical blood routine data; c) GSEA analysis results of different groups; d) Relative risks of blood routine panels corresponding to different diseases.

Our analysis applied a deep clustering algorithm to routine blood data from 160 participants, revealing three distinct physiological categories encompassing 80, 41, and 39 individuals each. The blood routine of each type of population can be distinguished, and the specific cluster analysis results and the probability density of blood routine data with significant differences are shown in **Figure** **3**a,b, with others shown in (Figure S1, Supporting Information). This stratification uncovers the underlying physiological diversity within the cohort, paving the way for deeper insights into disease risk profiles.

Subsequent transcriptome differential and Gene Set Enrichment Analysis (GSEA) for each category were performed and the enrichment of these genes highlighted their involvement in pathways linked to chronic diseases such as cancer, diabetes (Figure S2, Supporting Information). We further assessed disease risk disparities among the categories by comparing Enrichment Scores (ENS) for nine diseases in Figure 3c. The third group exhibited the highest ENS, indicating a higher risk for diseases such as Parkinson's disease, cardiomyopathy, and systemic lupus erythematosus. In contrast, the first and second groups displayed varying risk profiles for different diseases (Data S2, Supporting Information).

Additionally, we examined the blood phenotype characteristics of each category. Representative indices from each cluster's center were chosen to reflect the group's blood profile, with relative sizes of these indices across different risk categories shown in Figure 3d. In the high‐risk group, 25 routine blood indices typically presented larger values. Notably, for diseases like parkinsonism and systemic lupus erythematosus, the second group showed a moderate risk with a significant increase in basophilic granulocytes percentage (BAS%), which is crucial in the immune response and impacts these diseases. For other diseases, including leukemia and diabetes, the second group had a lower risk than the moderate‐risk first group, with a higher monocyte percentage (MON%) index underscoring its clinical relevance.

### 2.4 Multi‐Omics Classification Performance

To test the validity of multi‐omics classification methods, we conducted a comparative analysis of seven multi‐omics classification techniques, each employing a distinct method for processing complex multi‐omics data. These included the K‐nearest neighbor (KNN) classifier,^[^ ^40^ ^]^ Support vector machine (SVM) classifier,^[^ ^41^ ^]^ a Fully connected neural network classifier,^[^ ^42^ ^]^ Lasso regularization‐based classification,^[^ ^43^ ^]^ MOGONET (Multi‐Omics Graph Convolutional Networks),^[^ ^35^ ^]^ MOVE^[^ ^44^ ^]^ (Multi‐Omics Variational Autoencoders), and DeepIMV (A Variational Information Bottleneck Approach to Multi‐Omics Data Integration).^[^ ^45^ ^]^

**Figure 4:**
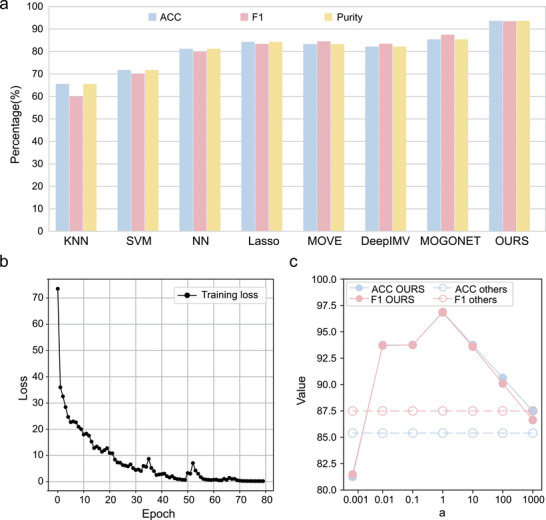
Comparison of classification results of multiple omics models and model loss convergence. a) The comparison between our proposed multi‐omics classification model and some mainstream multi‐omics models in ACC, F1, Purity. b) The convergence diagram of the model losses as the epoch changes. c) The comparison between the metrics of the model we proposed and the optimal metrics of other methods when the hyperparameter *a* changes within the range.

To evaluate the performance of these models, we focused on accuracy (ACC), F1 score, and purity, running each method ten times to determine the average results, as shown in **Figure** **4**a and Data S3 (Supporting Information). Our analysis, as evidenced in Figure 4a, reveals that our novel transformer‐based model outperforms other state‐of‐the‐art methods in multi‐group classification for supervised tasks. Remarkably, it demonstrates significant improvements over the second‐best algorithm across all three performance metrics, with mean increases of 8.3%, 6.0%, and 8.3%, respectively. This highlights the exceptional capability of our transformer‐based model in capturing complex interrelations within and between groups in omics studies, marking a significant advancement in the field of multi‐omics data analysis.

Additionally, we analyzed the convergence behavior of our model over iterative epochs, presented in Figure 4b, which indicated that it achieved stability after 40 epochs. We further conducted experiments to assess the impact of varying the hyperparameter *a*, as seen in Figure 4c. These experiments demonstrated that our method remained superior to other approaches under different conditions. This robust performance of our model underscores its efficiency and adaptability in multi‐omics data integration, establishing it as a valuable tool in multi‐omics research. In addition, detailed information about the ablation experiment of the model and specific parameter settings is shown Table S1–S4 (Supporting Information).

**Figure 5:**
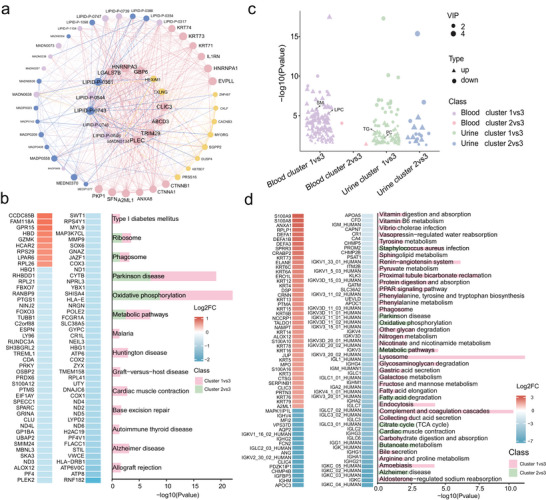
Identification and analysis of characteristic substances. a) Multi‐Omics data interaction network. b) Analysis of transcriptome differences between high and low risk groups. c) Analysis of metabolite differences between high and low risk groups. d) Analysis of protein differences between high and low risk groups.

A total of 79 transcriptomic, 79 proteomic, and 60 urinary and blood metabolomic features were identified as crucial histological features differentiating various risk groups (Data S4, Supporting Information). The enrichment analysis revealed that differential genes were mainly enriched in pathways related to bacterial infections, diabetes mellitus, and cancer pathogenesis. Differential proteins were associated with neutrophil degranulation and the negative regulation of protein modification processes, while metabolites were concentrated in glycerophospholipid metabolism and cancer pathogenesis pathways, as detailed in Supplementary Figure 3. The correlation analyses were performed to select substances with high inter‐group correlation. Using Cytoscape software, an interaction network of 56 key substances was analyzed. This analysis highlighted essential factors such as the *HEXIM* gene, implicated in AIDS, cardiac hypertrophy, and malignancies, and the TRIM29 protein, crucial in tumor‐related activities. In urinary metabolism, substances like PC, linked to neurodegenerative and liver diseases, and the blood metabolite TG, associated with cardiovascular risks and metabolic syndrome were identified in **Figure** **5**a. In order to further clarify the differences of substances in different groups, the difference analysis was made for substances in the highest and lowest groups according to the risk groups of different diseases. The different substances identified were shown in Figure 5b,c,d, and these different substances contain characteristic substances identified by deep learning models.

These findings demonstrate that significantly differentiated substances across cluster populations are involved in major regulatory pathways affecting various conditions, including cancers, metabolic diseases, and neurodegenerative disorders. In the progression of cancer, S100A9 and S100A8, as immune modulators, play key roles in tumor immune evasion and inflammation.^[^ ^46^ ^]^ Abnormal changes in metabolites such as TG, PC, and LPC are closely related to metabolic diseases (e.g., obesity, diabetes, non‐alcoholic fatty liver disease). Elevated TG levels are associated with insulin resistance, fat accumulation, and other metabolic disorders, while the role of PC in fat metabolism and cell membrane structure affects the progression of fatty liver and atherosclerosis. Abnormal PC metabolism has been found to be associated with metabolic reprogramming in cancer.^[^ ^47^ ^]^ These changes in metabolites not only reflect the pathogenesis of metabolic diseases but also provide potential molecular targets for metabolic regulation and targeted therapies. In neurodegenerative diseases, such as Alzheimer's disease, gene expression changes in FAM118A, CCDC85B, and FOXO3 are associated with apoptosis, neuroinflammation, and neuronal dysfunction. Disruption of FOXO3 may promote neurodegenerative processes by regulating oxidative stress responses and apoptotic pathways.^[^ ^48^ ^]^ A deeper understanding of these pathways helps elucidate the pathogenesis of neurodegenerative diseases. This study confirms the variability in disease risk, among groups classified based on blood test‐derived risk assessments, underscoring the value of multi‐omics data in disease risk stratification.

### 2.5 The Clinical Data Validation

**Figure 6:**
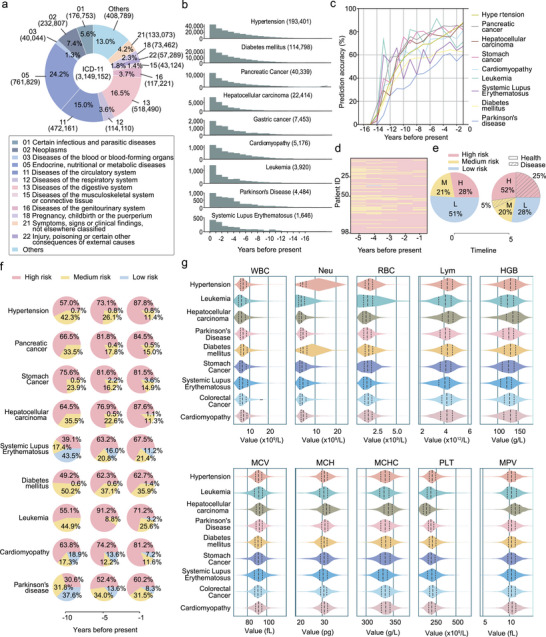
Large‐scale clinical data validation results. a) Distribution of clinical diagnoses by category; b) Routine blood sample size before diagnosis of various diseases over time; c) Disease prediction accuracy trends pre‐giagnosis; d) Heat map of risk changes of 100 hypertensive patients in the first five years before diagnosis; e, Risk and morbidity follow‐up of 100 people for 5 years; f) Pre‐diagnosis risk group proportion changes across different diseases; g) Distribution of important blood routine characteristics in different diseases.

To validate the clinical effectiveness of the proposed blood routine panel, we statistically analyzed data from 3 149 152 patients who visited the First, Fourth, and Ninth Medical Centers of the PLA General Hospital between 1993 and 2021, as shown in **Figure** **6**a. Our findings indicated that disorders of the endocrine, nutritional, and metabolic systems–particularly hypertension and diabetes mellitus–along with circulatory system diseases, were the most prevalent conditions in the studied cohort. Additionally, significant prevalence rates were observed for tumor diseases, such as gastric and liver cancer, highlighting the critical need to focus on these chronic conditions. Moreover, nine diseases were selected to show significant differences across population clusters. We meticulously analyzed the routine blood data of these patients, collected over 19 years preceding their diagnoses, totaling 393 631 data points. Figure 6b illustrates the sample sizes for these diseases across different time periods, providing insight into disease progression. Specific statistical indicators are shown in the Data S5 (Supporting Information), The probability density maps of blood indicators for each disease are shown in the Figures S8 and S9 (Supporting Information). Compared with the extensive changes in hematological parameters of individuals at high altitudes, the variation in blood indicators at low altitudes are small. An overall clustering analysis was performed on the clinical routine blood data over a 20‐year time series for nine diseases, classifying the routine blood data of each disease into distinct risk groups. The specific results are shown in Figure S4 (Supporting Information). Furthermore, we conducted an in‐depth analysis of routine blood data for each disease, segmenting it into different periods. We primarily focused on evaluating the proportions of high, medium, and low‐risk groups among patients at 1, 5, and 10 years before the onset of the diseases. The distribution of these risk groups is detailed in Figure 6c. In addition, we plotted the temporal change in the low‐risk group for the nine diseases, with results presented in Figure S5 (Supporting Information). To further validate our findings, we recruited 100 patients with hypertension and conducted a retrospective risk assessment based on their blood routine data for the first five years leading up to the time of diagnosis, with results shown in Figure 6d. Heat maps indicated a significant increase in the number of individuals at high risk as the diagnosis date approached. We also randomly selected 100 healthy individuals and conducted a follow‐up over a period of five years, as illustrated in Figure 6e. Our analysis demonstrated that as the proportion of high‐risk individuals increased, so too did the incidence of disease development, thereby supporting the predictive validity of our model. Furthermore, Figure 6f presents the proportion of individuals in the high‐risk group for each disease and their temporal changes preceding disease onset. These trends, including the increasing proportion of the high‐risk group as disease onset approached, were consistent with typical disease progression patterns, indicating the effectiveness of our blood test‐based risk grouping in predicting the emergence of nine chronic diseases. Specific values are provided in Data S6 and S7 (Supporting Information).

To further refine our analysis, we selected ten routine blood indicators commonly associated with chronic diseases: WBC, NEU, LYM, RBC, HGB, MCV, MCH, MCHC, PLT, and MPV. The distribution of these indicators across different patients is depicted in Figure 6g. Our statistical analysis revealed significant variations in the distribution of these clinical indicators among various chronic diseases. This diversity in index threshold values underscores the potential of clinical blood test indices as discriminative features for distinguishing between chronic diseases, thereby offering predictive insights into disease onset and providing a deeper understanding of chronic disease pathology and progression.

## 3 Discussion

In this work, we developed an analytical software leveraging transformer technology integrated with multi‐omics data for early warning of chronic diseases. This tool enables researchers to assess the risk of chronic conditions through a routine blood examination. We performed prospective validation using a large cohort from a major general hospital, analyzing blood test data collected over the past 20 years to predict the onset of nine cancer types, five cardiovascular diseases, and three mental illnesses. This method extends the traditional health management model by incorporating a big data management approach, thus offering a feasible solution for large‐scale chronic disease screening.

Previous research^[^ ^49^, ^50^, ^51^ ^]^ has primarily focused on the characterization of chronic disease states; However, accurately predicting pre‐disease states remains a formidable challenge. The onset of diseases is influenced by genetic and environmental factors, and NCDs may exhibit altered metabolic levels even before clinical symptoms arise. Therefore, comprehensive multi‐omics studies that integrate transcriptomics, proteomics, and metabolomics can reveal deeper connections within disease processes. Multi‐omics research has been widely applied in fields such as oncology and cardiovascular disease.^[^ ^52^, ^53^ ^]^ Early multi‐omics studies largely examined correlations among different omics layers, but with the continuous advancement of deep learning technologies, numerous cell analysis and disease landscape models utilizing transformers have emerged.^[^ ^54^, ^55^ ^]^ Notably, our study represents the first instance of translating multi‐omics findings into large‐scale clinical cohort applications. By combining multi‐omics results with existing NCD research, we employed deep learning capabilities, self‐attention mechanisms, and information fusion features to accurately identify pre‐disease states within populations while maintaining a degree of model interpretability.^[^ ^56^ ^]^

Currently, some novel biomarker detection methods based on artificial intelligence do not effectively predict the risk of tumor development.^[^ ^57^ ^]^ Routine blood tests remain one of the most basic and accessible laboratory assessments. Our multi‐omics analysis effectively differentiates risk warning values associated with routine blood test parameters across different disease states. Prior research has indicated that in the oncological field, parameters such as red blood cell distribution width (RDW), mean platelet volume (MPV), neutrophil/lymphocyte ratio (NLR), MPV/PC, and platelet count/lymphocyte ratio (PLR) can serve as adjunct tools to replace inflammatory markers in tumor diagnosis.^[^ ^58^, ^59^ ^]^ NLR, RDW, PLR, and MPV have also been implicated in the prediction of conditions such as hypertension and diabetes.^[^ ^60^, ^61^ ^]^ Furthermore, a strong correlation exists between hemoglobin levels and Parkinson's disease,^[^ ^62^ ^]^ with RDW also being associated with neurological disorders.^[^ ^63^ ^]^

We developed a user‐friendly and intuitive chronic disease risk prediction system interface, tailored for healthcare professionals and patients, facilitating easy access to health status and proactive preventive measures. The simplicity of this system substantially amplifies the impact of our findings, promoting wider adoption among healthcare settings. Importantly, our blood test offers a cost‐effective alternative for chronic disease risk prediction, with standard blood tests being less expensive and easily scalable across various healthcare settings. This approach has the potential to substantially reduce the healthcare costs associated with advanced chronic disease treatment and improve the prospects for early intervention. Another notable innovation of this study is the pioneering use of large‐scale electronic health record data. In the realm of neurological diseases, previous research has demonstrated the application of extensive electronic health data for predicting and assessing pre‐disease states.^[^ ^64^, ^65^ ^]^ Similarly, in oncology and cardiovascular fields, cohort studies have shown that serological indicators can serve as early predictors of disease onset.^[^ ^66^, ^67^ ^]^ However, integrating multi‐omics analysis with big data validation represents a novel approach in this context. We employed Enrichment Scores (ENS) to forecast the risk of various NCDs,^[^ ^68^ ^]^ effectively combining big data analytics with our testing methodology, which corroborated the model's high predictive value in real‐world settings.

Despite these significant achievements, our study has limitations. First, although the sample size is substantial, its applicability within community populations requires further validation. Second, while the model demonstrates promising performance, its interpretability could still be improved. Future research might focus on incorporating additional diagnostic markers and further refining machine learning methodologies to enhance both clarity and usability. Third, variations in genetic background, lifestyle, and treatment practices across populations could have some impact on the generalizability of the model. For example, the validation cohort primarily consisted of individuals from a single region with relatively uniform characteristics, which may not fully reflect the diversity of global populations.

Furthermore, as new data becomes available, the model will need regular updates to maintain its predictive accuracy and relevance. The heterogeneity of biochemical results was not incorporated into this study.

In summary, this research represents a significant step forward in personalized medicine and preventive health management. By combining standard blood test parameters with extensive multi‐omics data, we successfully developed an innovative blood test capable of predicting various chronic disease risks. This work substantially enhances the accuracy and efficiency of early disease diagnosis and provides broader public access to sophisticated medical predictive tools through the development of a chronic disease prediction platform. Despite limitations related to sample diversity and model interpretability, the results of this study undoubtedly lay a solid foundation for future research directions and clinical applications. As we explore more precise disease prediction models, we anticipate that these advancements will bring about revolutionary changes in global health management practices, particularly in the early detection and prevention of chronic diseases.

## 4 Conclusion 

In summary, our study demonstrates that a simple blood test can accurately predict chronic diseases and that our predictive model can be used to identify and implement early interventions to prevent or minimize the development of chronic diseases and their associated adverse health consequences. The dissemination of this approach will have far‐reaching implications for public health, particularly in terms of optimizing health management and reducing healthcare costs.

## 5 Experimental Section

### Overview

Our network, named “Omicsformer,” applies to semi‐supervised clustering of health data samples using a multi‐omics approach. This technique has four essential steps: (1) In the pre‐processing phase, irrelevant or repetitive data were removed to make the analysis more accurate. (2) Then applied a specialized learning technique, transformers, to each data type, such as urine and blood metabolomics, proteins, and genes. This helped to understand the unique characteristics of each data type. (3) The next crucial step involves merging these varied data forms into a single, comprehensive dataset. By using transformers' unique attention mechanism, this model can pay close attention to both the details within a single data type and the connections between different data types. (4) Finally, key substances were identified across these different data types using the trained model, pinpointing elements crucial for understanding complex health‐related pathways. The specific frame diagram is shown in Figure S6 (Supporting Information).

### Learning Process

In this section of this study, the learning process of the proposed method is elaborated, specifically designed for multi‐omics data analysis. It was commenced by considering multi‐omics data ${\left\{X^{\left(i\right)}\right\}}_{i=1}^{v}\in R^{n\times d^{\left(i\right)}}$, where *n* represents the number of samples and *v* denotes the number of distinct omics types. The main goal is to convert these high‐dimensional features into low‐dimensional representations for each omics type and integrate them into a cohesive analytical framework.

In the context of single‐omics data analysis, this transformer architecture is employed to generate low‐dimensional representations for each omics dataset. Specifically, for a given batch of single‐omics features **X** ^(*i*)^, the encoder was designed to produce corresponding query (**Q** ^(*i*)^), key (**K** ^(*i*)^), and value (**V** ^(*i*)^) matrices. The specific formula is as follows:

(1) $$Q^{\left(i\right)}=X^{\left(i\right)}W_{Q}^{\left(i\right)},\;K^{\left(i\right)}=X^{\left(i\right)}W_{K}^{\left(i\right)},\;V^{\left(i\right)}=X^{\left(i\right)}W_{V}^{\left(i\right)}$$

The learnable parameter matrices $W_{Q}^{\left(i\right)},W_{K}^{\left(i\right)},W_{V}^{\left(i\right)}$ were incorporated within this framework. In the self‐attention framework, the model is designed to calculate the degrees of relatedness, or weights, between each element in the sequence and all other elements. The formulation is as follows:

(2) $$H^{\left(i\right)}=\text{Self Attention}\;\left(Q^{\left(i\right)},K^{\left(i\right)},V^{\left(i\right)}\right)=\text{softmax}\left(\frac{Q^{\left(i\right)}{K^{\left(i\right)}}^{T}}{\sqrt{d_{H}^{\left(i\right)}}}\right)V^{\left(i\right)}$$

**H** ^(*i*)^ denotes the hidden representation of *i*‐th omics data from the attention transformation. $d_{H}^{\left(i\right)}$ denotes the dimensional size of the representation **H** ^(*i*)^. Given *v* omics, they were concatenated to generate **H** ^*mul*^ for multi‐omics integration.

(3) $$H^{mul}=\text{Concat}\left(H^{\left(1\right)},\text{…},H^{\left(v\right)}\right)$$

### Multi‐Omics Information Integration

In our approach to multi‐omics integration, first generated a multi‐omics hidden representation, denoted as **H** ^*mul*^. This representation is subsequently input into the multi‐omics transformer module. To facilitate this process, we initialize the multi‐omics parameter matrices $W_{Q}^{mul},W_{K}^{mul}$, and $W_{V}^{mul}$ for the generation of attention vectors, which can be mathematically expressed as:

(4) $$Q^{mul}=H^{mul}W_{Q}^{mul},\;K^{mul}=H^{mul}W_{K}^{mul},\;V^{mul}=H^{mul}W_{V}^{mul}$$

It is important to note that the dimensional size of the concatenated representation **H** ^*mul*^ varies with the number of omics involved. To ensure uniformity and facilitate fair comparison across different datasets, the multi‐omics transformer module was employed to reduce the dimensions of **H** ^*mul*^ to a fixed size *d* ^(*mul*)^. Consequently, the final multi‐omics integration representation **H** ^*F*^ is computed as follows:

(5) $$\begin{array}{c} H^{F}=\text{Attention}\left(Q^{mul},K^{mul},V^{mul}\right)=\text{softmax}\left(\frac{{Q^{mul}K^{mul}}^{T}}{\sqrt{d^{\left(mul\right)}}}\right)V^{mul} \end{array}$$

This model architecture diverges from the traditional symmetric encoder‐decoder structure, opting instead for a process focused on dimensional reduction. A method of iteratively compressing and reducing dimensions in the transformer module is used to mitigate noise or unreliable data in the original omics.

### Optimization

A two‐phase training mechanism was implemented to optimize the model's performance in the actual training process. This approach involves an initial training round, followed by a subsequent fine‐tuning phase.

In the first training phase, pre‐training the model parameters for each omics type by reconstructing the original data was considered. This process is essential to ensure that the model's optimization is not aimless but rather guided by a clear objective. The reconstruction of each omics data ${\hat{X}}^{\left(i\right)}$ is achieved through a decoder function *g*, which maps the latent space representation **H** ^(*i*)^ back to the input space. The decoder function is defined as g(★):H↦X, where ${\hat{X}}^{\left(i\right)}$ is the reconstructed data of the *i*‐th omics. The reconstruction loss, which is key to the pre‐training phase, is formulated as follows:

(6) $$L_{re}=\sum_{i=1}^{v}{\left\|{\hat{X}}^{\left(i\right)}-X^{\left(i\right)}\right\|}_{F}^{2}$$

In the second phase, the training process was refined by incorporating classification loss alongside the Mean Squared Error (MSE) loss. Specifically, CrossEntropyLoss was introduced, which is instrumental in guiding the optimization of learned representations. The CrossEntropyLoss is calculated as:

(7) $$L_{Cross}=\frac{1}{n_{tra}}\sum_{p=1}^{n_{tra}}{\left(y_{p}-{\hat{y}}_{p}\right)}^{2}$$

where *n*_*tra*_ represents the number of training set samples, *y*_*p*_ denotes the true label of the *p*‐th sample, and ${\hat{y}}_{p}$ represents the predicted label of the *p*‐th sample. In this context, ${\hat{y}}_{p}$ in real training is a distribution processed by the softmax function, representing the probabilities of the different classes. Consequently, the final loss function is formulated as:

(8) $$L=L_{MSE}+a*L_{Cross}$$

with *a* being a hyperparameter. Upon completing the optimization of the model and obtaining reliable sample representations, proceeded to compute and mine the underlying relationships within the multi‐omics data. This comprehensive approach ensures not only the fidelity of the model in reconstructing and classifying the omics data but also its capability to uncover and interpret complex biological interconnections.

### Important Substances and Pathways

The elucidation of key molecules and pathways within multi‐omics data is pivotal for integrating multi‐omics information and gaining insights into individual health status across varying levels. This methodology adopts an innovative approach to feature importance evaluation. It involves systematically nullifying each column signal in the preprocessing phase and scrutinizing the consequent effects on the model's performance. This technique allows to ascertain the significance of specific features in classification tasks by observing the extent to which the model's performance deteriorates upon their removal. This approach, commonly employed in neural networks, is particularly effective in prioritizing and ranking crucial molecules within multi‐omics datasets. The process of identifying important molecules and their contributions in each omics group through this technique is methodical and involves several key steps: (1) Post‐Learning Feature Nullification: After the model completes its learning phase, we sequentially set the features of multi‐omics data to zero during the training process. (2) Performance Metrics Observation: Then monitored the model's performance metrics post multi‐omics integration after the alteration of each feature. These metrics were compared against the baseline metrics obtained when all molecules are included. Through this comparison, the molecules were identified that result in the most significant decrease in performance metrics within each omics group. (3) Identification of Key Molecules: Molecules that cause the greatest decline in the model's performance are deemed the most important within the multi‐omics dataset. This study uses the F1 macro metric to assess the model's performance. Ten independent experiments were conducted on the model to minimize the impact of random variations. This approach ensures robustness in the selection of molecules that consistently demonstrate the largest decrease in performance across these experiments. Finally, a correlation analysis of these important molecules were conducted across multiple omics groups. This analysis is instrumental in uncovering key pathways interlinking these molecules, thereby providing a comprehensive understanding of their interplay and collective impact within the multi‐omics context. This approach not only highlights the critical molecules but also sheds light on the complex molecular interactions and pathways that underpin different health statuses.

### Sample Preparation and Extraction

The experiment and recruitment criteria were carefully designed and a total of 160 healthy Chinese residents were recruited, all participants were of similar age, with a mean age of 25 years, all participants adhered to uniform living and dietary arrangements, participants resided in employer‐provided dormitories, ensuring similar living conditions. All meals were provided by the same employer‐operated cafeteria, where participants consumed traditional Chinese cuisine, including rice, wheat, vegetable oil, meat, and fish. This standardized diet minimized dietary variability, which is a common confounder in microbiome and metabolomics studies. They were healthy, had no known clinical illnesses, and did not take medications or receive interventions during the test. See Table S1 (Supporting Information) for raw data statistics. In the morning when participants were fasting, 8 mL of anticoagulated blood was collected using EDTA‐K2, mixed by gentle up and down mixing, draw 3 mL of whole blood from the EDTA‐K2 collection vessel, total RNA was extracted utilizing TRIzol R LS Reagent (Thermo Fisher Scientific, Inc, Waltham, MA, USA), according to the manufacturer's protocol. Draw another 1.5 mL of whole blood from the EDTA‐K2 collection vessel, centrifuged using a centrifugation speed of 4000 rpm for 10 min, and take 200ul supernatant and put them into 1.5 mL centrifuge tubes. Total RNA collected from 160 individuals was sent to UW Genetics (https://www.bgi.com) for RNA sequencing. The supernatant was taken for metabolite assays, all of which were performed by Metware Biotechnology (Wuhan, China). In the morning while fasting, participants collected midstream urine samples, with each person collecting 50–100 mL for proteomic analysis. The urine samples were centrifuged at 1500 rpm for 10 min, and after separating the supernatant, 200 µ*L* of each sample was placed into 1.5 mL centrifuge tubes. The proteomic analysis was carried out by iCarbonX in Shenzhen. Furthermore, routine blood testing was carried out at the PLA General Hospital, with all blood tests and their basic statistical descriptions comprehensively outlined in Table S1 (Supporting Information). To preserve the integrity of each sample, they were transferred to −80°C storage within 20 min of collection. This prompt and precise handling was crucial to maintaining the quality of the samples, ensuring the reliability and accuracy of the subsequent omics analyses.

### Sequencing Analysis

Sequencing analysis was conducted meticulously to ensure the highest data quality. The preparation of RNA libraries was carried out using the MGIEasy RNA kit. Total RNA was utilized for library construction, adhering to the protocols specified for the BGISEQ‐500 platform. The sequencing itself was executed on the BGISEQ‐500 platform. For alignment purposes, the resultant FASTQ sequencing files were aligned to the GRCh38 human genome using the STAR aligner, with a specific focus on retaining uniquely mapped reads for subsequent analyses.^[^ ^69^ ^]^

Metabolite analysis was performed employing the Metware Metabolic Assay System. This involved initial preparation and extraction of liquid samples. Hydrophilic compounds were processed using 20% acetonitrile methanol internal standard extracts, followed by centrifugation to obtain the supernatant. Conversely, hydrophobic compounds underwent extraction via MTBE: MeOH. Both hydrophilic and hydrophobic compounds were then subjected to separate analyses using Ultra Performance Liquid Chromatography (UPLC). The UPLC conditions for hydrophilic compounds encompassed T3 and Amide, while hydrophobic compounds were analyzed using a C30 column. Following the UPLC analysis, hydrophilic and hydrophobic compounds were examined using an ESI‐Q TRAP‐MS/MS mass spectrometry system. The metabolite data were acquired through Linear Ion Trap (LIT) and Triple Quadrupole (QQQ) scanning modes, providing an exhaustive overview of the samples' composition and abundance of metabolites.

For the proteomic analysis, protein samples were extracted from 160 urine samples using a specialized ultracentrifugation method. This process included sample recovery, centrifugation, addition of peptide lysis buffer, enzymatic digestion, and separation of tryptically digested peptides. The peptides were isolated using a homemade capillary column fitted with C18 particles and analyzed through an UltiMate 3000‐HPLC system (Thermo Fisher Scientific). Mass spectrometry was conducted using a Thermo Fisher Orbitrap mass spectrometer, coupled with the aforementioned HPLC system. Stringent quality control measures were enforced throughout the mass spectrometry testing process. These measures ensured that all samples met the protein identification number density profile criteria, with at least 80% of the 1000 proteins being tested in line with a 30‐min gradient for the prepared samples. The results of the samples used for subsequent data analysis adhered to these quality control requirements, ensuring that the outcomes were both accurate and reliable.

### Analytical Methods

The filtered count values were converted to the trimmed mean of M‐values (TMM)‐normalized transcripts per million mapped reads (FPKM) values by DESeq2 package.^[^ ^70^ ^]^ Further analysis was performed using proteins with a coefficient of variation <0.3 and missing data frequency less than 0.25. Logarithmic transformation (log2) and mean centering on all metabolic and protein data were performed. The network analysis was conducted with Cytoscape software.^[^ ^71^ ^]^ Gene Ontology biological process (GOBP) and Kyoto Encyclopedia of Genes and Genomes (KEGG) enrichment analyses of the feature substances were measured by deep learning methods.^[^ ^72^ ^]^ The redundant GO terms were removed using the simplifying function (by = “p.adjust,” cutoff = 0.3). The statistical significance of the GO enrichment was tested using Benjamini and Hochberg with a cutoff *q* value < 0.05. All the KEGG analyses with *p* values < 0.05 were enriched and shown. For mapping the metabolites and transcripts into pathways, Pathview^[^ ^73^ ^]^ was used to produce detailed mapping for the selected pathways. All statistical analyses and article mapping were performed using R (version 4.0.2). Additional data processing, first performed missing data imputation on the omics datasets. For features with a missing rate lower than 20%, missing values were imputed by replacing them with the minimum non‐zero value of the dataset divided by 1/83. Furthermore, the data were standardized using the R function scale, which centers each variable by subtracting its mean and scales it to unit variance.

### Blood Routine Clustering and Clinical Validation Methods

Routine blood parameters serve as quintessential biomarkers, reflecting the health status of individuals. This study aims to apply unsupervised learning techniques to cluster routine blood test data. This approach is designed to unveil the intrinsic structures within these parameters, facilitating the stratification of disease risk. Given the routine blood data $B\in R^{n\times d^{B}}$, our initial step is to map this data into a potential space using a multi‐attention mechanism. This process aims to discern potential rules within the data in a high‐dimensional space, as described by the following equation:

(9) $$H^{B}=\text{softmax}\left(\frac{BW_{Q}^{B}{\left(BW_{K}^{B}\right)}^{T}}{\sqrt{d^{HB}}}\right)BW_{V}^{B}$$

In this equation, $W_{Q}^{B}$, $W_{K}^{B}$, and $W_{V}^{B}$ are learnable parameters, and *d* ^*HB*^ is the dimension of **H** ^*B*^. To learn the high‐dimensional representation of **B**, a decoder was employed to reconstruct the routine blood data and optimize the parameters of the entire network, as indicated by:

(10) $$L_{B}={∥B-g(H^{B})∥}_{F}^{2}$$

Optimizing the above equation results in the representation **H** ^*B*^ in the latent space, from which labels were derived through clustering. This methodology allows for the extraction of high‐order information embedded within routine blood data. Subsequent clustering of this refined data yields representations for each cluster, encapsulating the risk stratification of various patient demographics.

Cluster centroids are computed by consolidating representations from all samples within a cluster, with their mean serving as the representative centroid of each respective risk level. In the clinical validation of the model, routine blood test data were obtained from a patient database, corresponding to their health status prior to illness onset. Euclidean distances between these clinical samples and the derived cluster centroids are computed to assign a risk category to each patient. Proximity to a centroid is indicative of the corresponding risk level. As visualized in Figure 3c, the indicators' relative magnitudes were normalized and illustrated across the three risk‐centric clusters. This validation also extends to a longitudinal analysis. Here, the sample distribution over each year leading up to the onset of the disease is statistically scrutinized. This analysis is pivotal in substantiating our model's efficacy by observing variations in the proportion of individuals classified within high‐risk categories as they approach the critical juncture of disease manifestation. This study aims to provide a robust and nuanced understanding of disease risk stratification through this multi‐faceted approach, combining sophisticated machine learning techniques with rigorous clinical validation. This methodological framework not only enhances our ability to predict disease risk but also offers valuable insights into the temporal dynamics of risk factor evolution, contributing significantly to the field of predictive health analytics.

### Criteria for the Selection of the Validation Cohort

To ensure the suitability and quality of the validation cohort, strict inclusion and exclusion criteria were established. For inclusion criteria, only samples that met the diagnostic standards and had complete clinical blood routine data were included. This ensured that all samples used for validation were clinically relevant and suitable for assessing the predictive performance of the model. For exclusion criteria, samples were excluded if they had incomplete blood routine data, exhibited significant data abnormalities, or involved patients with severe comorbidities that could confound the analysis. These criteria were designed to maximize the reliability of the validation results by reducing noise and ensuring data quality.

## 6 Webserver Construction

In our endeavor to enhance accessibility to advanced health analytics, we have developed a web server specifically tailored for users to utilize our model for predicting disease risk based on routine blood data. The web server is hosted at [http://riskprediction.xyz/ ], designed to be user‐friendly and informative. The architectural design and processing logic of the web server are shown in (Figure S7, Supporting Information). The server's homepage delineates the underlying principles of our predictive model, coupled with medical advice from experienced doctors. Users seeking to understand their disease risk can easily navigate to the “Start Analysis” page. Here, they can either manually input their blood routine data or upload their data files for analysis. Our model is adept at predicting risks for nine common diseases, making it a versatile tool in personal health management. Upon clicking and submitting their data, the server analyzes the user's blood routine data. This process is executed on the server side, ensuring efficient and accurate computation. Subsequently, the server presents the user with predicted risk results and professional recommendations from doctors affiliated with the PLA General Hospital. This dual output of predictive data and expert advice offers a comprehensive understanding of the user's health status. To further enhance user experience and comprehension, we have incorporated features to visualize both the blood routine data and the predicted results. This visualization enables users to easily interpret their health status, assisting them in making timely and informed decisions regarding medical consultations or treatments. Recognizing the growing reliance on mobile technology, our platform is fully compatible with mobile devices. Users can effortlessly access the web server on their smartphones, upload their blood routine data, and receive the same detailed analysis and prediction level as on a desktop. This mobile compatibility significantly expands the accessibility of our service, allowing users to engage with our platform from virtually anywhere. And we've developed a simple user manual for easy use, hyperlinked to: https://github.com/dzboop/user‐menu.

Our system has been validated with a large volume of clinical data, further establishing its reliability and accuracy. Additionally, we have integrated personalized medical advice to help high‐risk patients improve their health status. These recommendations are tailored to provide practical guidance for individual health management. In the future, we plan to continuously enhance the system to foster stronger connections between patients and doctors, making the system more human‐centric and improving the overall patient experience. Our commitment to this project is rooted in the desire to create a convenient, efficient, and user‐centric disease risk prediction platform. We aim to empower individuals with actionable health insights, bridging the gap between routine blood tests and proactive health management. Through this platform, we strive to contribute meaningfully to the field of personalized medicine and public health.

## 7 Study Approval

All study protocols were approved by the ethics committee of the Chinese PLA General Hospital with the approval identifier (ID) S2019‐035‐01 and were in accordance with established national and institutional ethical guidelines. The study was clearly described to all participants, who signed informed consent forms before the collection of blood and personal information.

## Conflict of Interest

All authors declare no competing interests.

## Author Contributions

Z.D., P.L., Y.J., and Z.W., contributed equally to this work. K.H., D.Z., P.L., and K.G. conceived the idea behind this study. H.C., X.Z., C.L., C.Z., Q.Z., C.R., and D.W. were responsible for the collection and processing of multiple omics and clinical data. Z.W., S.W., J.G., S.F., S.L., and D.H. were responsible for analyzing the data. Z.D., Y.J., P.L., X.L., and E.Z. were responsible for writing the first draft. All the authors contributed to the final manuscript.

## Supporting information

Supporting Information

Supporting Information

## Data Availability

The data that support the findings of this study are available from the corresponding author upon reasonable request.
