# Supplementary material for: Integrative Multi‐Omics and Routine Blood Analysis Using Deep Learning: Cost‐Effective Early Prediction of Chronic Disease Risks
Source: Adv Sci (Weinh). 2025 Apr 2;12(22):2412775. doi: 10.1002/advs.202412775 (PMC12165040; doi:10.1002/advs.202412775)
Supplement: Supplementary file 1 — Supporting Information [file ADVS-12-2412775-s002.pdf]

ADVANCED SCIENCE
Open Access

Supporting Information

for *Adv. Sci.*, DOI 10.1002/advs.202412775

Integrative Multi-Omics and Routine Blood Analysis Using Deep Learning: Cost-Effective Early Prediction of Chronic Disease Risks

Zhibin Dong, Pei Li, Yi Jiang, Zhihan Wang, Shihui Fu, Hebin Che, Meng Liu, Xiaojing Zhao, Chunlei Liu, Chenghui Zhao, Qin Zhong, Chongyou Rao, Siwei Wang, Suyuan Liu, Dayu Hu, Dongjin Wang, Juntao Gao, Kai Guo*, Xinwang Liu*, En Zhu* and Kunlun He**

Supplementary Figures And Tables

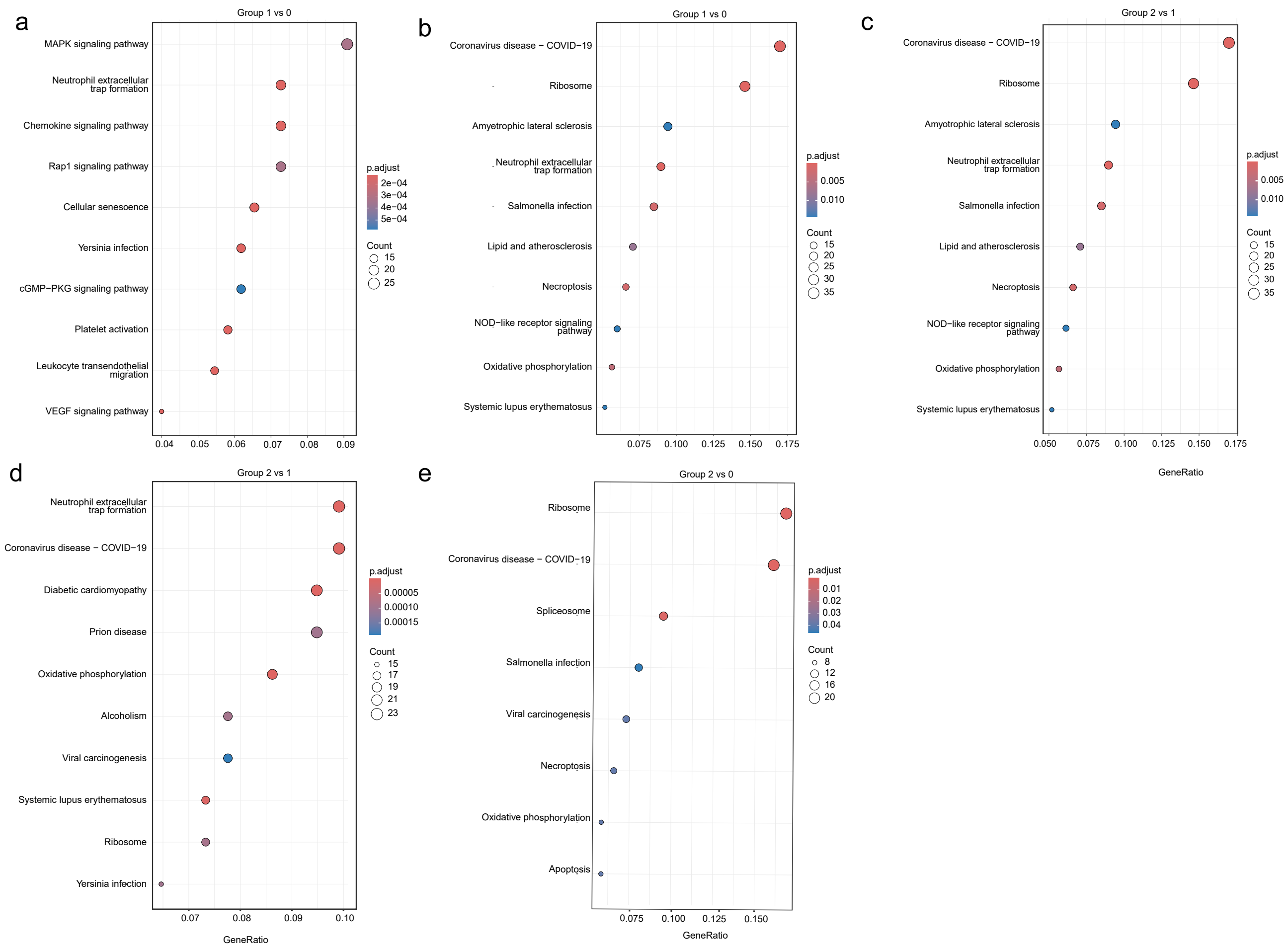

Figure S1: The enrichment result for each category.

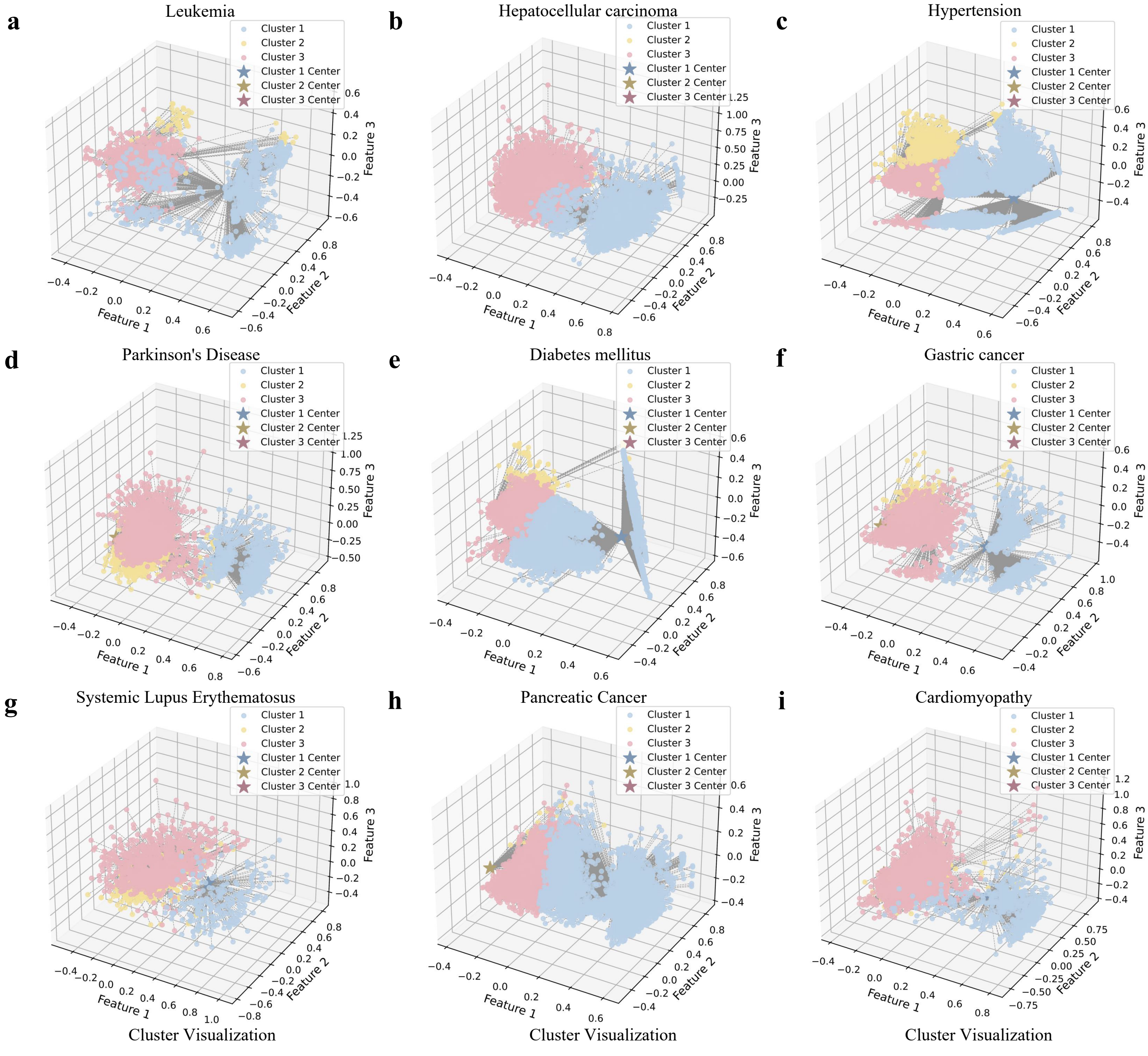

Figure S2: Visualization of clustering results for different diseases.

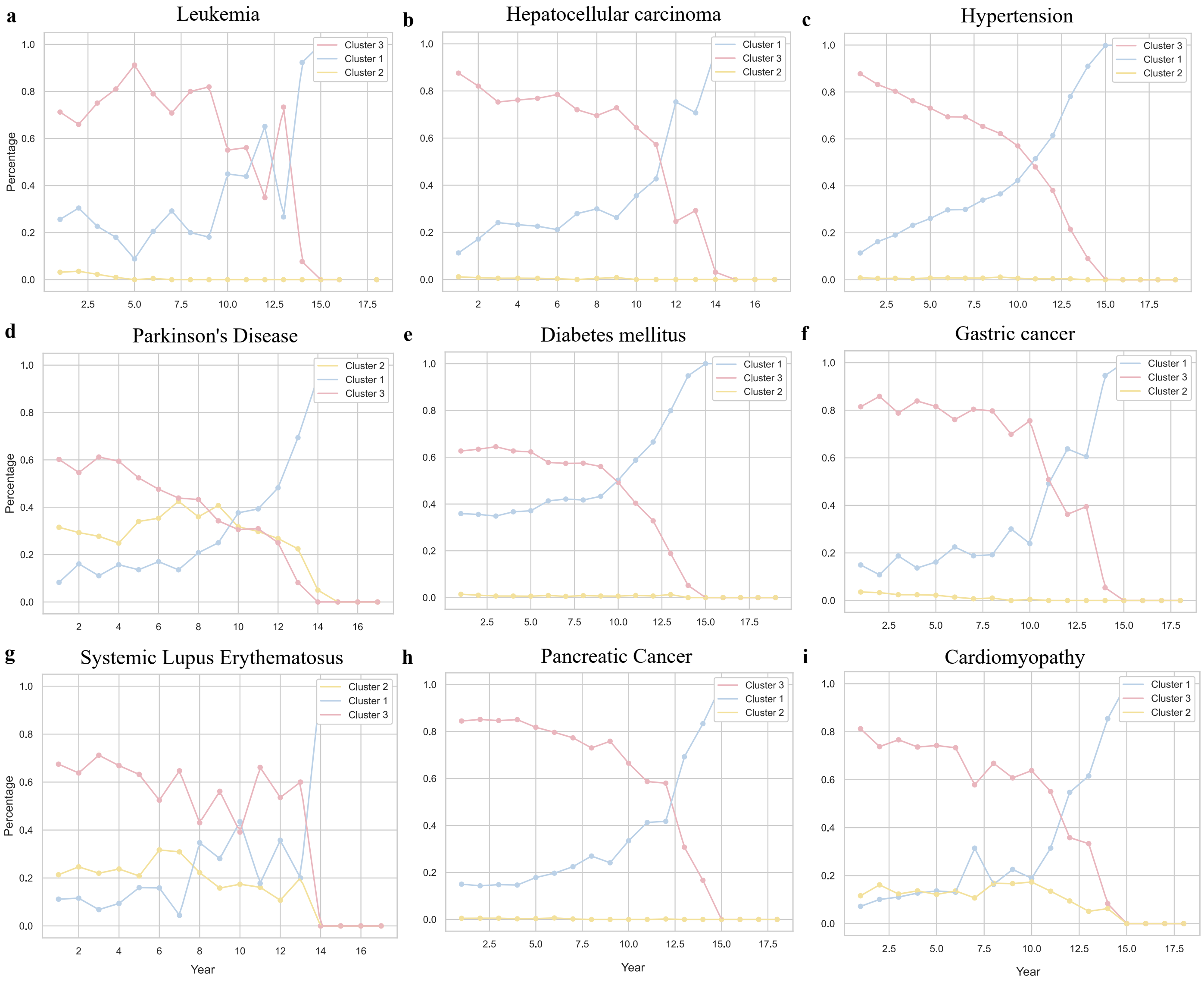

Figure S3: Visualization of changes in risk over time for different diseases.

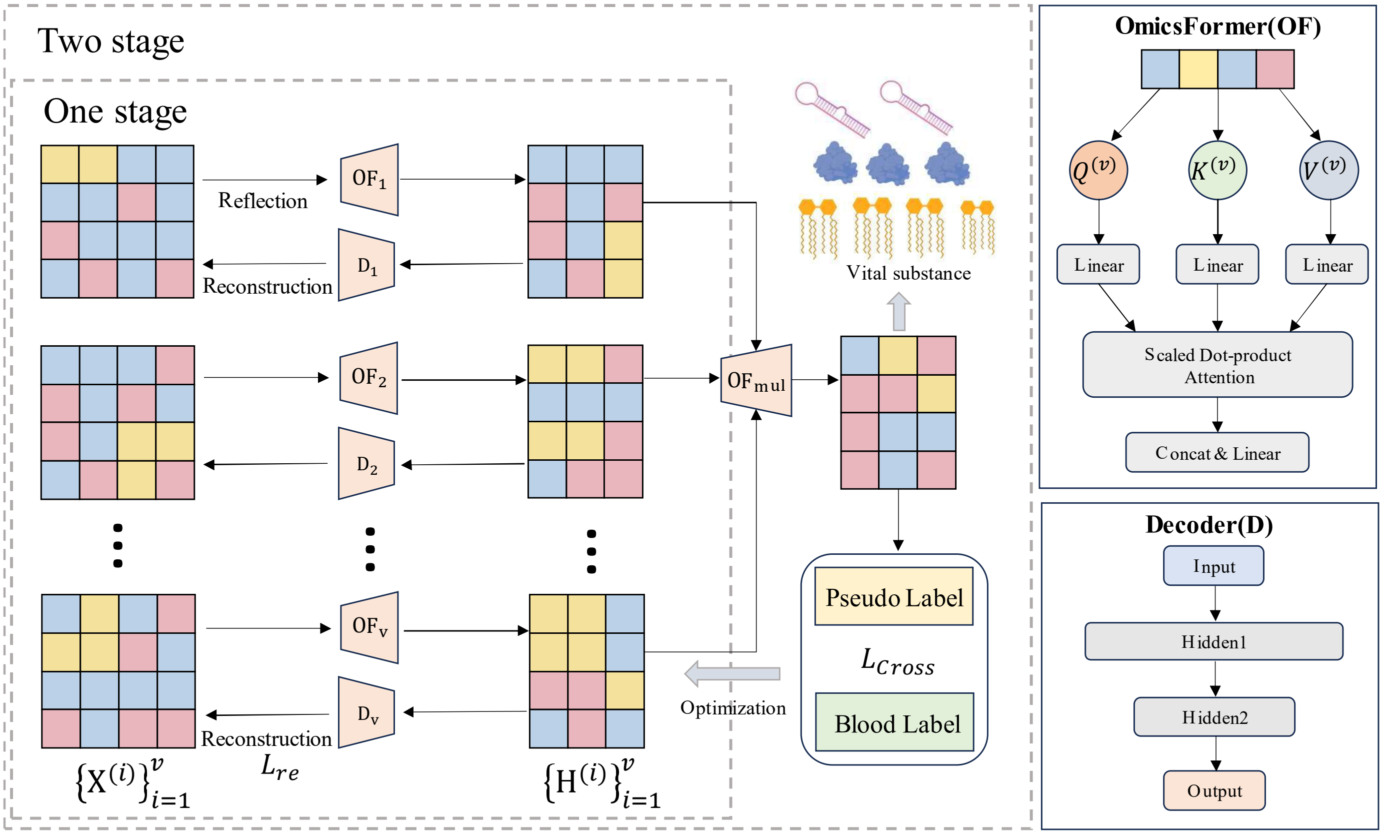

Figure S4: The multi-omics network framework.

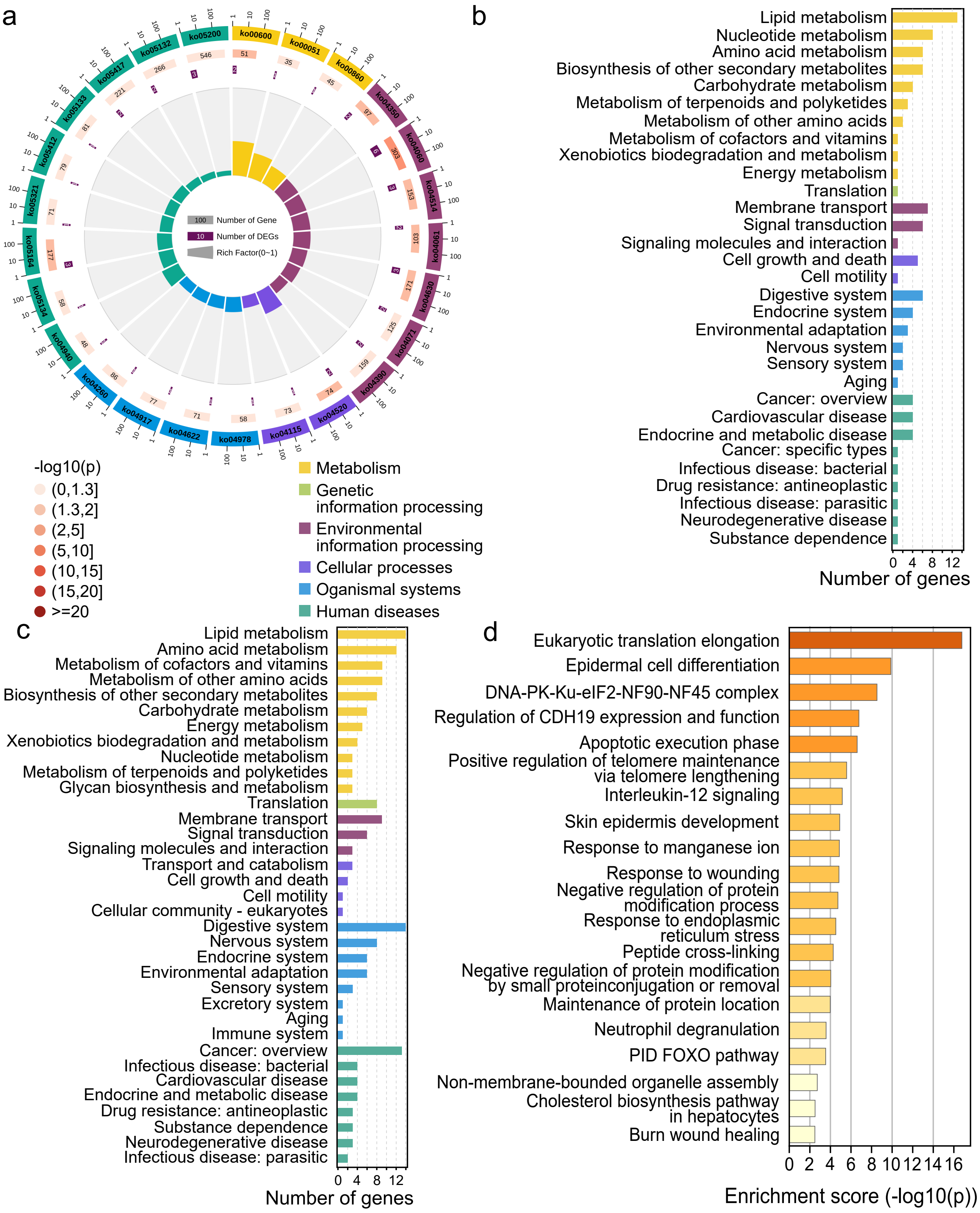

Figure S5: **Multi-omics sigmlicant feature analysis.** a, Functional enrichment analysis of diferentially expresed genes (DEGs); b, KEGG pathway annotation ofsienilicant diierential metaboltes in blod. c,KEGG pathway annotation ofsienif-cant diterential metabolites in uine: d, Pathway ennchmen analysis from protein data.

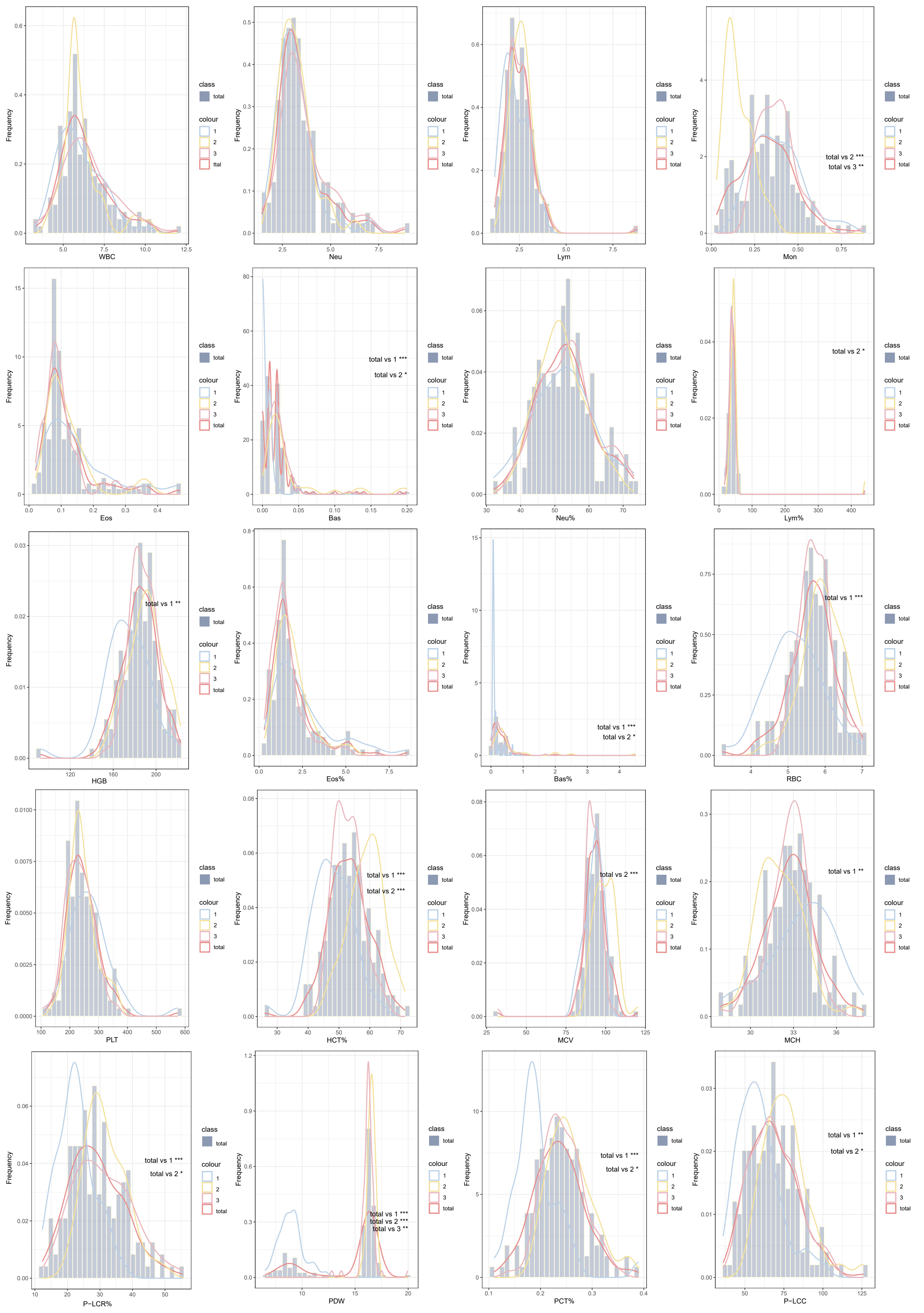

Figure S6: Blood routine distribution of plateau population.

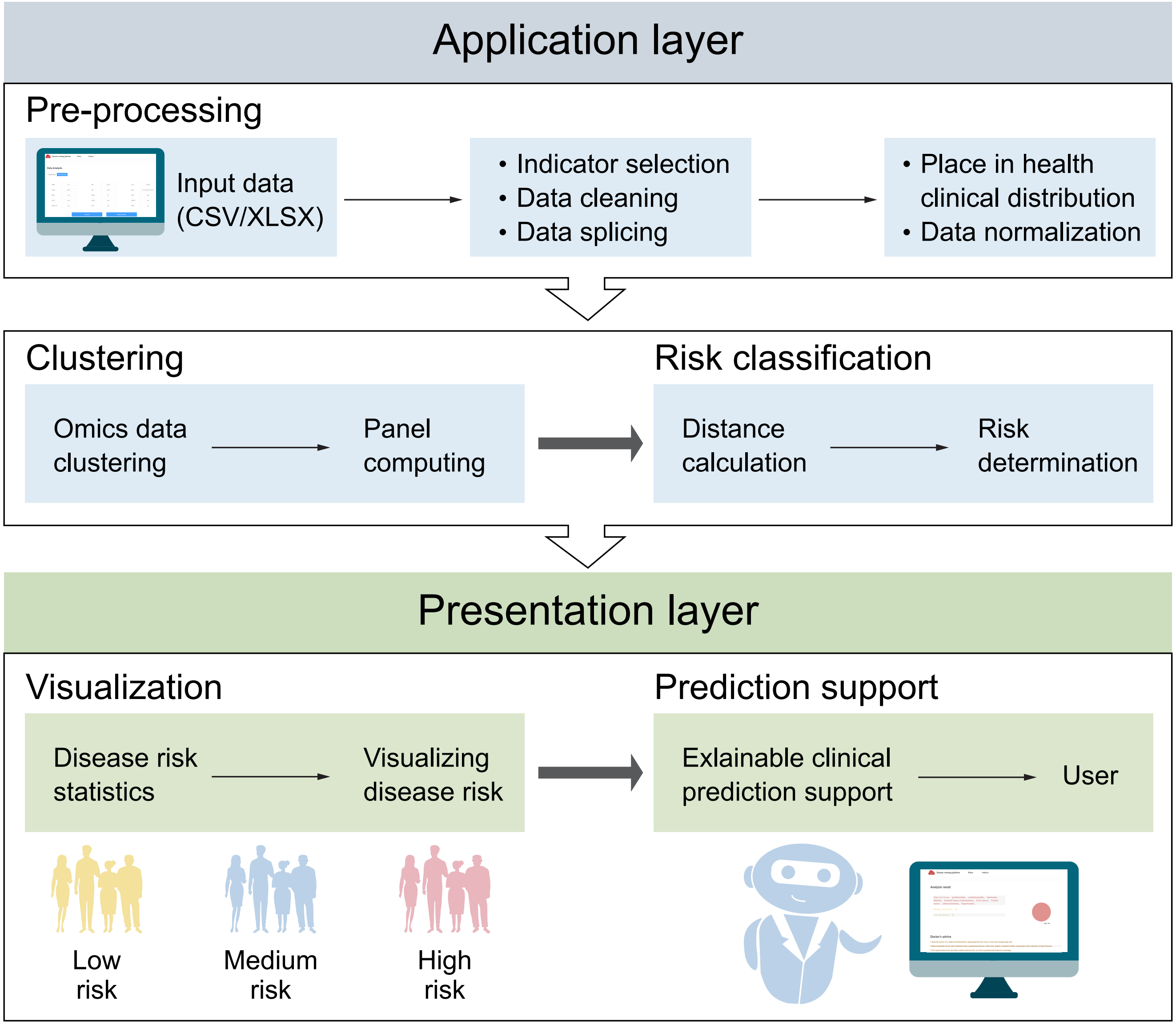

Figure S7: Web server architecture design and processing logic.

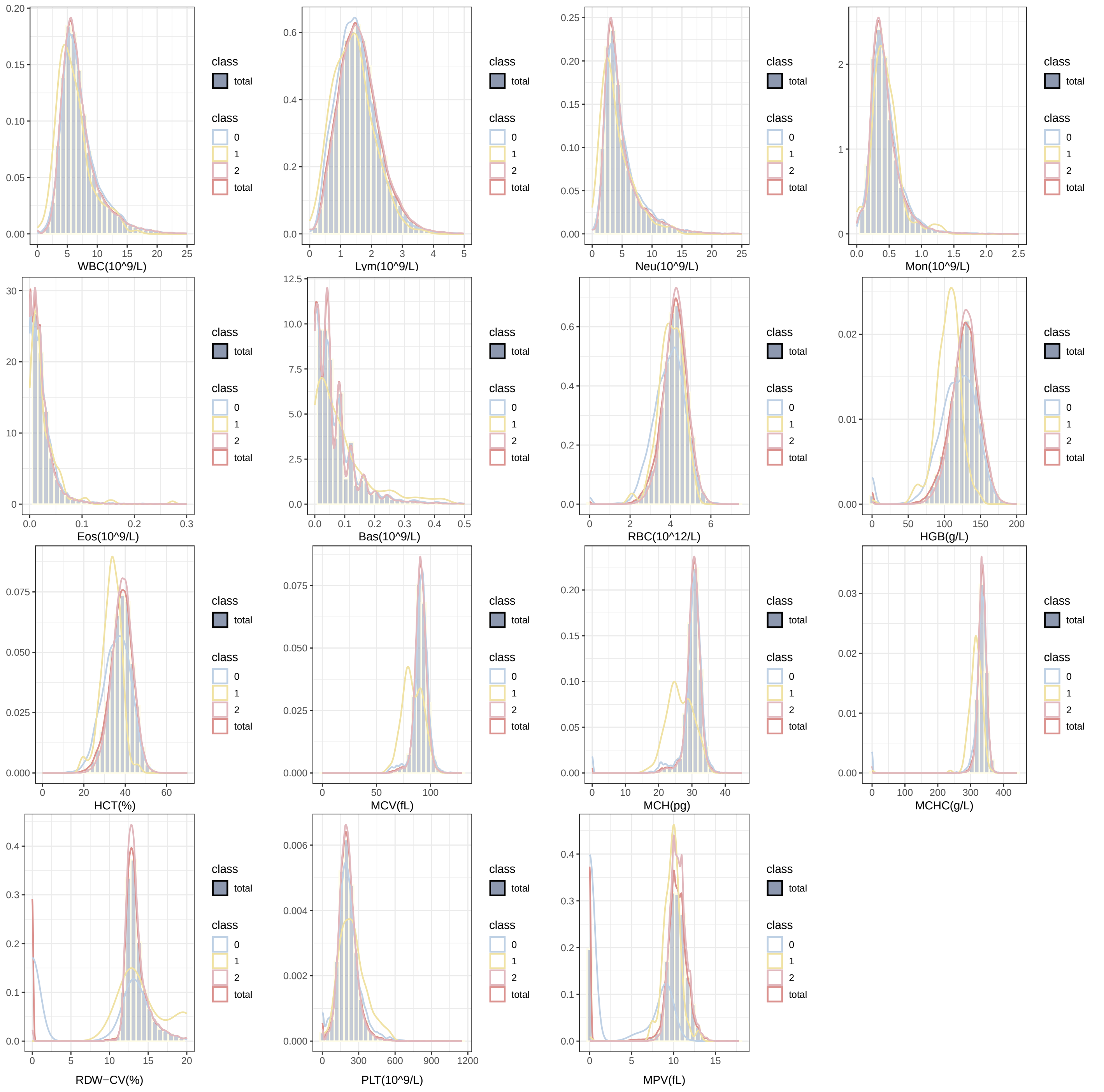

Figure S8: Blood routine distribution of adenocarcinoma patients.

APPENDIX TABLE

Table S1: Pre-training and supervision module ablation.

Components	Metrics(%)			
	ACC	NMI	Purity	F-score
Raw data	43.75	4.30	51.88	45.09
Pre-training	64.06	25.53	64.84	64.98
Omicsformer	**96.88**	**89.14**	**96.88**	**96.84**

Table S2: Multi-Head Attention Feature Extraction module ablation.

Strategies	Metrics(%)			
	ACC	NMI	Purity	F-score
Autoencoder	46.88	4.89	50.00	47.61
Multi-Head Attention	**96.88**	**89.14**	**96.88**	**96.84**

Table S3: Multi-Omics Information Aggregation module ablation.

Strategies	Metrics(%)			
	ACC	NMI	Purity	F-score
Urine metabolomics	78.12	49.04	78.12	77.89
Blood metabolomics	84.38	64.07	84.38	84.59
Proteomics	68.75	41.78	68.75	68.63
Transcriptomics	84.38	59.50	84.38	84.26
Multi-Omics Aggregation	**96.88**	**89.14**	**96.88**	**96.84**

Table S4: Parameter Settings.

Parameter	dim	batch size	pre-epoch	aline-epoch	lr-pre	lr-aline
Value	128	16	200	50	0.0005	0.0001

Table S5: Variable Definitions.

Variable Name	Unit	Variable Name	Unit
White blood cell Count (WBC)	10^9/L	Neutrophil Percentage (Neu%)	-
Neutrophil Count (Neu)	10^9/L	Lymphocyte Percentage (Lym%)	-
Lymphocyte Count (Lym)	10^9/L	Monocyte Percentage (Mon%)	-
Monocyte Count (Mon)	10^9/L	Eosinophil Percentage (Eos%)	-
Eosinophil Count (Eos)	10^9/L	Basophil Percentage (Bas%)	-
Basophil Count (Bas)	10^9/L	Red Blood Cell Count (RBC)	10^12/L
Platelet Large Cell Count (P-LCC)	10^9/L	Platelet Large Cell Ratio P-LCR(%)	-
Hemoglobin Concentration (HGB)	g/L	Red Cell Distribution Width-Coefficient of Variation (RDW-CV(%))	-
Hematocrit (HCT(%))	-	Red Cell Distribution Width - Standard Deviation (RDW-SD)	fL
Mean Corpuscular Volume (MCV)	fL	Platelet Count (PLT)	10^9/L
Mean Corpuscular Hemoglobin (MCH)	pg	Mean Platelet Volume (MPV)	fL
Mean Corpuscular Hemoglobin Concentration (MCHC)	g/L	Platelet Distribution Width (PDW)	fL
